# Traumatic head injuries in children: demographics, injury patterns, and outcomes in Saudi Arabia

**DOI:** 10.1186/s12245-024-00808-w

**Published:** 2025-01-02

**Authors:** Hussin Albargi, Rayan Jafnan Alharbi, Ateeq Almuwallad, Naif Harthi, Yahya Khormi, Hari Krishnan Kanthimathinathan, Sharfuddin Chowdhury

**Affiliations:** 1 Programme of Emergency Medical Service, College of Nursing and Health Science, Jazan University, Al Maarefah Rd, Jazan, 45142 Saudi Arabia; 2 Department of Surgery, Faculty of Medicine, Jazan University, Jazan, Saudi Arabia; 3 Department of Neurosurgery, King Fahd Central Hospital, Jazan, Saudi Arabia; 4 Birmingham Children’s Hospital, Birmingham, UK; 5 Trauma Centre, King Saud Medical City, Riyadh, Saudi Arabia; 6 School of Health Professions, University of Alabama at Birmingham, Birmingham, AL USA

**Keywords:** Children head injury, Trauma, Mortality, Public health

## Abstract

**Background:**

Traumatic head injuries (THIs) are among the leading cause of mortality and intensive care unit (ICU) admission in children worldwide. Most of the published literature concerning THIs arises predominantly from North America and Europe. However, only limited data about the incidence, characteristics and impact on children in Saudi Arabia exists.

**Methods:**

We conducted a retrospective analysis of THIs in children (≤ 18 years of age) using data from the Saudi TraumA Registry (STAR) from August 2017 to December 2022. Data included patient demographic characteristics, the mechanism, type and severity of injury. We used multivariable logistic regression to assess the association between outcomes and clinical factors.

**Results:**

We identified 466 children with THI. Most children were over six years of age (69.5%) and male (76.6%). Motor vehicle crashes (MVCs) were the most common cause of THIs (51.9%), with falls being more common in infants (69.8%). Over half of the children required ICU admission. Children with higher injury severity score, heart rate at presentation to the ED, hospital stay duration, respiratory assistance and need for surgery were more likely to require ICU admission. The overall mortality rate was 7.7%, with schoolchildren (age: 6–12 years) having the highest mortality rate (10.8%). Higher rates of ICU admission were associated with increases in the injury severity score (ISS), hospital stay duration, respiratory assistance and the need for surgery.

**Conclusions:**

Children in the 6–12 year age-group had the highest mortality rate, reflecting high injury severities associated with increased ICU admissions. These findings highlight the importance of targeting preventive measures for MVCs in older children and improving trauma care for severe cases.

**Clinical trial number:**

Not applicable.

## Background

Globally, traumatic injuries are a major cause of morbidity and mortality across all age groups [1, 2]. In children, traumatic head injuries (THIs) are responsible for over 80% of trauma-related deaths [3–5]. THIs are more pronounced in young children due to their unique anatomy. Proportionally, infants and young children have a large head compared to their body size and a less developed bone structure that provides less cranial protection and increases the risk of serious injuries [6]. As children grow and become mobile, they become more vulnerable to different types of traumatic injuries, such as road traffic accidents (RTAs). In Saudi Arabia, RTAs are associated with prolonged hospital stays and high mortality rates, highlighting a significant challenge that needs to be addressed [7, 8].

Differences in mechanism and severity of head injuries may exist based on different paediatric age groups. In infants and young children, falls are common and can cause severe head injuries, even from short distances [9, 10]. In contrast, head injuries in older children are often caused by high-impact trauma events such as RTAs, bicycle accidents and sports injuries [11]. Skull fractures are common in infants, likely due to falls being the most frequent cause of head injuries in this age group. However, in older children, the injury severity varies depending on the type of injury experienced. Concussions may occur after sport-related injuries, while severe brain injuries are more likely after RTAs and falls [12]. Therefore, understanding how factors influencing the severity of head injuries contribute to critical outcomes, such as intensive care unit (ICU) admission and mortality, may contribute to initiatives to improve care.

Traumatic injuries can be classified based on severity (minor, mild, moderate or severe). The Glasgow Coma Scale (GCS) is a measurement tool that focuses on the neurological status of patients suffering from head injuries [13]. The scores range from 3 (coma) to 15 (normal), with GCS scores classified as 13–15 for mild, 9–12 for moderate, and 3–8 for severe THI. In contrast, the injury severity score (ISS) is a more comprehensive measurement tool that includes multiple organ injuries, including the head. The scores range from 1 (minor) to 75 (critical) [14]. Studies have found that severe head injuries are associated with longer hospital stays and more ICU admissions [7, 14, 15]. The mortality rate in cases with severe injuries is higher than those with mild or moderate injuries [16]. Several factors may play a crucial role in patient outcomes after paediatric head injuries, including demographic characteristics, the mechanism of injury and injury severity. Majority of the published literature on trauma and outcomes are from the Western hemisphere, particularly North America and Europe. However, there is limited information regarding the association of these factors and outcomes in children in Saudi Arabia. Therefore, this study aimed to compare the demographic and injury characteristics of paediatric patients with THIs and assess factors associated with ICU admissions and mortality rates.

## Methods

This was a retrospective cohort analysis of prospectively collected data at King Saud Medical City (KSMC) in Saudi Arabia from August 2017 to December 2022, using the Saudi TraumA registry (STAR). KSMC is a level 1 trauma centre with over 1,400 beds, including 200 ICU beds and 100 emergency department (ED) beds. The STAR registry includes traumatic events affecting all ages and collects demographic, clinical and outcome data to improve trauma care in Saudi Arabia [17]. Details about the STAR registry have been previously described [18]. This study included children under 18 years of age who had a THI and met STAR inclusion criteria from August 2017 to December 2022. We included all children with THI and suspicion of a brain injury at the point of presentation to the ED. These included children with polytrauma (suspicion of a head injury and other limb or organ injuries), as well as those who were only eventually identified after assessment to have had a superficial head injury, without a brain injury, such as those with scalp haematoma at a later stage. All patients with suspected head injuries had brain CT scan or pan CT scan for polytrauma or intubated patient as a hospital protocol.

We used the following age-groups during analysis (Infant < 1 year, Preschool 1–6 years, Schoolchildren 6–12 years and Adolescents 13–18 years). The characteristics of the injury included: the mechanism of injury (motor vehicle includes both drivers and passengers, motorcycle, pedal cyclist, pedestrian, fall > 1 m, fall ≤ 1 m, assault/accidents and other); the time of the injury based on work and school start and end times (day: 6:00 am to 4:59 pm; night: 5:00 pm to 5:59 am); the location where injury occurred, i.e. home, on the road or at school; and the care mode of arrival, which was categorised as Red Crescent ambulance, helicopter, government ambulance, private ambulance or private/police vehicle. The prehospital and hospital interventions included procedures at the scene, trauma team activation, blood transfusions in the ED and whether the patient received respiratory assistance (unassisted respiration, which means breathing room air, and assisted respiration, which refers to any assistance from supplemental oxygen to a definitive airway, such as intubation). The type of injury was classified as skull fracture, brain contusion, intracranial haematoma, scalp injuries, and other brain injuries such as diffuse axonal, nerve, and brain stem injuries. In children with multiple injury types, the worst head injury was considered as injury type during the analysis. Vital sign variables included the first systolic blood pressure (BP), first heart rate (HR), first respiratory rate (RR), and first O2 saturation at the definitive care for hospital course. Treatment variables included days in the ICU, hospital stay duration and whether patients required disposition from the ED. Based on GCS at the time of presentation to the ED we grouped level of consciousness into the following injury severity categories: minor (13–15), moderate (9–12) and severe (3–8). The ISS results were categorised into minor (≤ 15), moderate (16–25), severe (26–40) and critical (> 40). We included surgery for any reason including for example, fixation of fractures in patients with polytrauma as a surgical procedure in the analysis. The primary outcome was in-hospital mortality, and the secondary outcome was ICU admission.

### Statistical analysis

Categorical variables are reported as frequencies and percentages, and continuous variables as medians and interquartile ranges (IQRs). The chi-squared test was used to examine differences between the categorical variables. Fisher’s exact test was used when the sample was small. The Kruskal-Wallis and the Mann-Whitney tests were used for continuous data. A Kaplan-Meier survival analysis was performed to estimate time-to-event outcomes (mortality). The log-rank test was used to compare mortality across GCS and ISS categories. We used univariate logistic regression to calculate the unadjusted odds ratio (OR) with a 95% confidence interval (CI) to report the association between ICU admissions and factors such as age, gender, injury location, mechanism of the injury, injury type, mode of arrival, trauma activation, blood transfusion, BP, HR, respiratory assistance, duration of hospital stays and the need for surgery. Multivariable logistic regression was used to identify the adjusted odds ratio (aOR), including factors that showed an association at the univariate level, a p-value threshold of 0.1 was used for inclusion in the final model. Statistical significance was defined as *p* < 0.05. All statistical analyses were conducted using Stata 16.0 (Stata Corp., College Station, TX, USA).

## Results

A total of 466 paediatric patients with head injuries were included in the study. The baseline characteristics in the paediatric age groups are detailed in Table 1. Most cases were in the school children group, with 37.7% of the total cases, followed by the adolescent group, with 31.7% of cases. Approximately three-quarters of the cases were males (76.7%). Most of the THIs occurred during night-time, and motor vehicle crashes (MVCs) were the prevalent cause of injury (75.5% and 51.9%, respectively). Scalp injuries were the most common (48.7%). Over 80.0% of cases occurred on the road, and two-thirds were transported via government ambulance (65.0%). Most patients had not undergone any procedure at the scene (86.3%), and respiratory assistance (including during inter-facility transfer) was administered in fewer than half of the patients (46.0%).

The overall GCS median was 15 (IQR 7–15), with a slight decrease in the adolescent group, indicating higher brain injury severity than the other age groups (median 12.5, IQR 7–15). As age increased, the median ISS increased, again reflecting increased injury severity in older children (Table 2). More than half of the children required ICU admission (56.2%), and the overall in-hospital mortality was 7.7% (*n* = 36).

**Table 1:** Demographic and clinical presentation characteristics of paediatric traumatic head injury patients

Variables	Total	Infant	Preschool	Schoolchildren	Adolescent	*P* value
	*N*= 466	*N* = 43	*N*= 99	*N* = 176	*N* = 148	
Gender *n* (%)	**466 (100)**	**43**	**99**	**176**	**148**	< 0.001
Male	357 (76.6)	31 (72.1)	67 (67.7)	127 (72.2)	132 (89.2)	
Female	109 (23.3)	12 (27.9)	32 (32.2)	49 (27.8)	16 (10.8)	
Nationality *n* (%)	**460 (98.7)**	**42**	**98**	**174**	**146**	< 0.001
Saudi	353 (76.7)	36 (85.7)	73 (74.5)	119 (68.4)	125 (85.6)	
Non-Saudi	107 (23.3)	6 (14.3)	25(25.5)	55 (31.6)	21 (14.4)	
Injury time, *n* (%)	**466 (100)**	**43**	**99**	**176**	**148**	
Day	114 (24.5)	26 (60.5)	27 (27.3)	40 (22.7)	30 (20.3)	
Night	352 (75.5)	17 (39.5)	72 (72.7)	136 (77.3)	118 (79.7)	
Mechanism of injury, *n* (%)	**466 (100)**	**43**	**99**	**176**	**148**	< 0.001
MVC	242 (51.9)	11 (25.6)	32 (32.3)	98 (55.7)	101 (68.2)	
Motorcycle	24 (5.2)	0 (0)	0 (0)	10 (5.7)	14 (9.4)	
Pedal	5 (1.1)	0 (0)	0 (0)	0 (0)	5 (3.4)	
Pedestrian	66 (14.2)	1 (2.3)	24 (24.2)	30 (17.0)	11 (7.4)	
Fall > 1 m	62 (13.3)	6 (14.0)	26 (26.3)	22 (12.5)	8 (5.4)	
Fall ≤ 1 m	44 (9.4)	24 (55.8)	8 (8.1)	10 (5.7)	2 (1.4)	
Assault/Accident	15 (3.2)	1 (2.3)	5 (5.1)	4 (2.3)	5 (3.4)	
Other	8 (1.7)	0 (0)	4 (4.0)	2 (1.1)	2 (1.4)	
Injury type *n* (%)	**466 (100)**	**43**	**99**	**176**	**148**	0.004
Skull fracture	43 (9.2)	7 (16.3)	15 (15.2)	14 (7.9)	7 (4.7)	
Brain contusion	62 (13.3)	5 (11.6)	15 (15.2)	17 (9.7)	25 (16.9)	
Intracranial haematoma	103 (22.1)	16 (37.2)	24 (24.2)	35 (19.9)	28 (18.9)	
Scalp injuries	227 (48.7)	12 (27.9)	43 (43.4)	96 (54.5)	76 (51.4)	
Other brain injuries	31 (6.7))	3 (7.0)	2 (2.0)	14 (8.0)	12 (8.1)	
Injury location, *n* (%)	**406 (87.1)**	**33**	**85**	**154**	**134**	< 0.001
Home	68 (16.8)	23 (69.7)	29 (34.1)	14 (9.1)	2 (1.5)	
Road	333 (82.0)	10 (30.3)	56 (65.9)	136 (88.3)	131 (97.8)	
School	5 (1.2)	0 (0)	0 (0)	4 (2.6)	1 (0.7)	
Definitive care mode of arrival, n (%)	**452 (96.9)**	**43**	**99**	**176**	**148**	< 0.001
Red Crescent	43 (9.5)	1 (2.3)	4 (4.3)	19 (11.2)	19 (13.0)	
Helicopter	13 (2.9)	2 (4.7)	1 (1.1)	4 (2.3)	6 (4.1)	
Government	294 (65.0)	21 (48.8)	57 (61.3)	117 (68.8)	99 (67.8)	
Private ambulance	24 (5.3)	2 (4.7)	4 (4.3)	9 (5.3)	9 (6.2)	
Private/police vehicle	78 (17.3)	17 (39.5)	27 (29.0)	21 (12.4)	13 (8.9)	
Procedure at the scene, *n* (%)	**466**	**43**	**99**	**176**	**148**	0.003
Yes	64 (13.7)	1 (2.3)	6 (6.1)	32 (18.2)	25 (16.9)	
No	402 (86.3)	42 (97.7)	93 (93.9)	144 (81.8)	123 (83.1)	
Trauma team activation, *n* (%)	**465 (99.7)**	**43**	**99**	**175**	**148**	0.55
Yes	78 (16.8)	4 (9.3)	16 (16.2)	31 (17.7)	27 (18.2)	
No	387 (83.2)	39 (90.7)	83 (83.8)	144 (81.8)	121 (81.8)	
Blood transfusion in ED, *n* (%)	**466**	**43**	**99**	**176**	**148**	0.25
Yes	50 (10.7)	4 (9.3)	14 (14.1)	13 (7.4)	19 (12.8)	
No	416 (89.3)	39 (90.7)	85 (85.9)	163 (92.6)	129 (87.2)	
On arrival at the ED, median (IQR)						
First systolic BP		101 (96–112)	107 (100–120)	110 (100–121)	121 (110–133)	< 0.001
First heart rate		132 (118–149)	122 (110–136)	111 (96–129)	94 (83–110)	< 0.001
First respiratory rate		24 (20–30)	24 (20–26)	20 (20–23)	20 (18–20)	< 0.001
First O_2_ saturation		97 (96–99)	99 (97–100)	99 (98–100)	99 (98–100)	0.022
Respiratory assistance, *n* (%)	**459 (98.5)**	**42**	**98**	**172**	**147**	0.005
Assisted respiration	211 (46.0)	11 (26.2)	37 (37.8)	87 (50.6)	76 (51.7)	
Unassisted respiration	248 (54.0)	31 (73.8)	61 (62.2)	85 (49.4)	71 (48.3)	

BP, blood pressure; ED, emergency department; IQR, interquartile range; MVC, Motor vehicle crash

**Table 2:** Level of consciousness, injury severity, and patient outcome following injury events

Variables	Total	Infant	Preschool	Schoolchildren	Adolescent	*P* value
	*N*= 466	*N* = 43	*N*= 99	*N* = 176	*N* = 148	
GCS						0.01
Median (IQR)	15 (7–15)	15 (15–15)	15 (7–15)	15 (7–15)	12.5 (7–15)	
GCS score, *n* (%)	**404 (86.7)**	**33**	**88**	**153**	**130**	0.07
3 − 8	147 (36.4)	5 (15.1)	31 (35.2)	59 (38.5)	52 (40.0)	
9 − 12	28 (6.9)	2 (6.1)	4 (4.6)	9 (5.9)	13 (10.0)	
13 − 15	229 (56.7)	26 (78.8)	53 (60.2)	85 (55.6)	65 (50.0)	
ISS						< 0.001
Median (IQR)	17 (9–22)	9 (9–18)	14 (9–21)	17 (9–24)	17 (13–25)	
ISS group, *n* (%)	**466 (100)**					0.011
≤ 15	214 (45.9)	29 (67.4)	53 (53.5)	80 (45.6)	52 (35.1)	
16–25	161 (34.6)	11 (25.6)	32 (32.3)	58 (32.9)	60 (40.5)	
26–40	77 (16.5)	3 (6.9)	10 (10.0)	32 (18.2)	32 (21.6)	
> 40	14 (3.0)	0 (0)	4 (4.0)	6 (3.4)	4 (2.7)	
Disposition from ED, *n* (%)	**466 (100)**					0.141
Ward	201 (43.1)	27 (62.8)	47 (47.5)	69 (39.2)	58 (39.2)	
ICU	207 (44.4)	10 (23.3)	39 (39.4)	82 (46.6)	76 (51.4)	
Operating theatre	52 (11.2)	5 (11.6)	12 (12.1)	21 (11.9)	14 (9.4)	
Mortuary/died in ED	5 (1.1)	1 (2.3)	1 (1.0)	3 (1.7)	0 (0)	
Discharged home	1 (0.2)	0 (0)	0 (0)	1 (0.6)	0 (0)	
Requires surgery, n (%)	**466 (100)**					< 0.001
Yes	222 (47.6)	11 (25.6)	37 (37.4)	86 (48.9)	88 (59.5)	
No	244 (52.4)	32 (74.4)	62 (62.6)	90 (51.1)	60 (40.5)	
ICU admission, *n* (%)	**466 (100)**					0.006
Yes	262 (56.2)	14 (32.6)	53 (53.5)	104 (59.1)	91 (61.5)	
No	204 (43.8)	29 (67.4)	46 (46.5)	72 (40.9)	57 (38.5)	
Days in ICU (in days)	**466 (100)**					
Median (IQR)	2 (0–11)	0 (0–5)	2 (0–10)	3 (0–11)	3 (0–13)	0.037
Days in hospital (in days)						
Median (IQR)	7 (4–19)	5 (2–14)	6 (4–18)	7 (4–17)	10 (5–25)	0.01
Discharge Destination *n* (%)	**466 (100)**					0.07
Discharged home	399 (85.6)	42 (97.7)	86 (86.9)	148 (84.1)	123 (83.1)	
Transfer	31 (6.7)	0 (0)	7 (7.1)	9 (5.1)	15 (10.1)	
Mortuary/died	36 (7.7)	1 (2.3)	6 (6.1)	19 (10.8)	10 (6.8)	
Mortality	**466 (100)**					0.19
Yes	36 (7.7)	1 (2.3)	6 (6.1)	19 (10.8)	10 (6.8)	
No	430 (92.3)	42 (97.7)	93 (93.9)	157 (89.2)	138 (93.2)	

ED, emergency department; GCS, Glasgow Coma Scale; ICU, intensive care unit; ISS, injury severity score; IQR, interquartile range

**Fig. 1:**
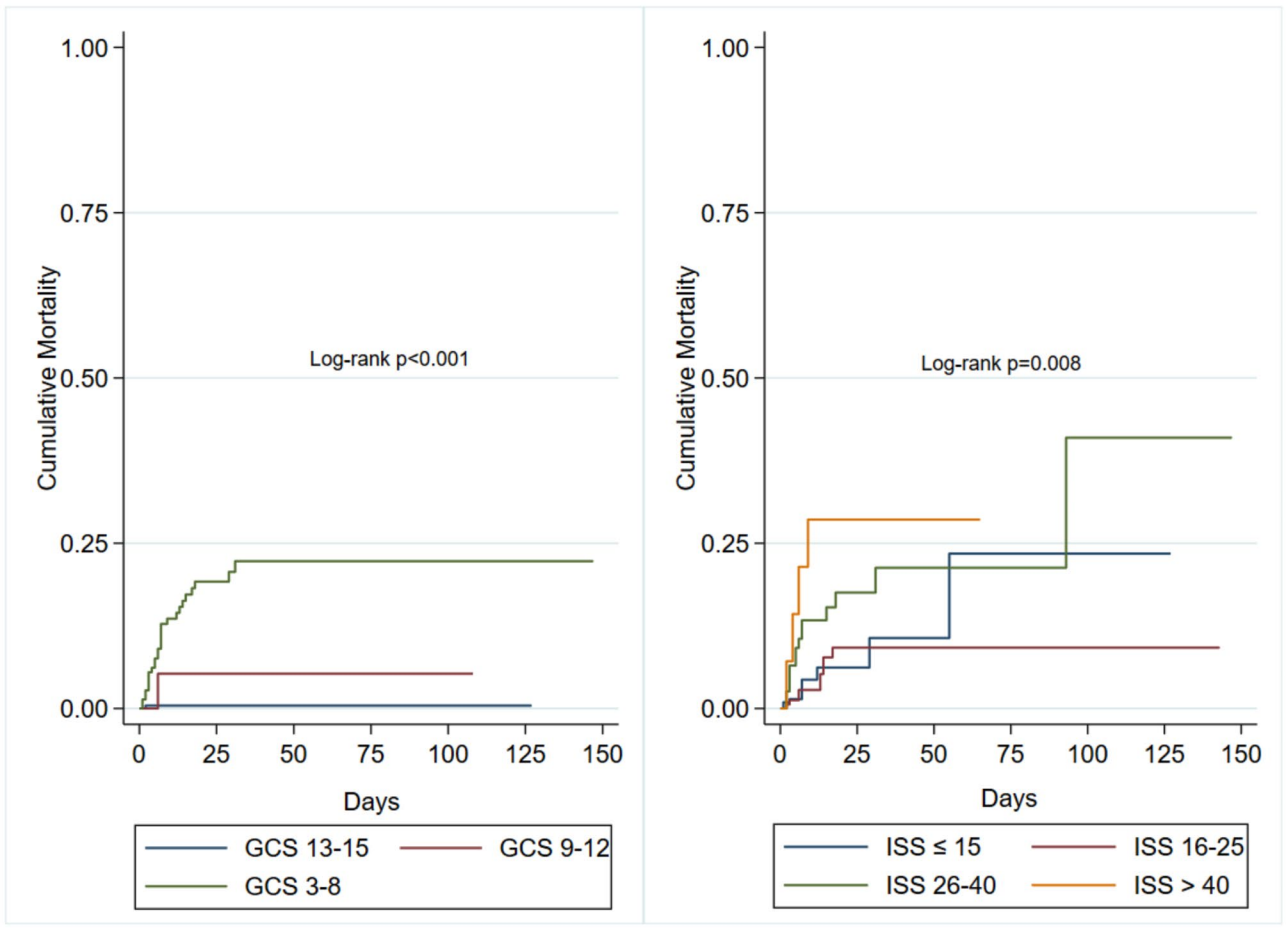
Kaplan-Meier failure estimates for GCS categories (left) and ISS categories (right). Left: Cumulative mortality for GCS 3–8 (green), GCS 9–12 (red), and GCS 13–15 (blue), with a log-rank test result of *P* < 0.001. Right: Cumulative mortality for ISS ≤ 15 (blue), ISS 16–25 (red), ISS 26–40 (green), and ISS > 40 (orange), with a log-rank test result of *p* = 0.008. GCS, Glasgow Coma Scale; ISS, injury severity score

Table 3 presents the variables and outcomes by in-hospital mortality status. Lower GCS scores and a higher ISS were strongly associated with increased mortality (*p* < 0.001). When the GCS scores and the ISSs were categorised, the Kaplan-Meier curves revealed higher mortality in children with severe injuries based on lowest GCS group (GCS: 3–8) and the highest ISS group (> 40) (Log-rank test: p 0.001, 0.008, respectively) Fig. 1. In cases where a trauma team was activated, the patient received a blood transfusion or had respiratory assistance were associated with greater mortality (*p* < 0.001)0.83.3% of children who died received ICU care prior to death, with other deaths occurring in either ED or during surgery prior to ICU admission. Children who died were more likely to have had shorter hospital stays and ICU admission, (*p* < 0.001).

In the univariate analysis, infants were less likely to be admitted to the ICU than adolescents (OR 0.30, 95% CI 0.14–0.62). However, after adjusting for the mechanism of injury, injury type, mode of arrival, ISS, trauma team activation, blood transfusion, respiratory assistance, length of hospital stay and need for surgery, the association between infants and ICU admissions was not significant (aOR 0.56, 95% CI 0.12–2.46). In the multivariable logistic regression analysis, motorcycle accidents (aOR 5.24, 95% CI 1.29–21.2), fall from ≤ 1 m (aOR 5.01, 95% CI 1.28–19.5) and assaults/accidents (aOR 18.6, 95% CI 3.26–106.3) were more likely to result in an ICU admission than MVCs. There was a 6.0% increase in the odds of an ICU admission for each unit increase in the ISS (aOR 1.06, 95% CI 1.02–1.11). Additionally, ICU admission was associated with respiratory assistance (aOR 25.9, 95% CI 11.5–58.4), length of hospital stay (aOR 1.14, 95% CI 1.08–1.19) and needing surgery (aOR 3.11, 95% CI 1.49–6.51) Table 4.

**Table 3:** Variables and outcomes by mortality status

Variables and outcomes	Total *N* = 466	Mortality		*P* value
		Survived *N* = 430	Died *N* = 36	
GCS score, Median (IQR)	15 (7–15)	15 (7–15)	3 (3–6)	< 0.001
GCS score, *n* (%)	**404**	**375**	**29**	< 0.001
− 8	147 (36.4)	120 (32.0)	27 (93.0)	
9 − 12	28 (6.9)	27 (97.2)	1 (3.5)	
13 − 15	229 (56.7)	228 (60.8)	1 (3.5)	
ISS Median (IQR)	17 (9–22)	17 (9–22)	26 (15–31)	< 0.001
ISS, *n* (%)	**466 (100)**	**430**	**36**	< 0.001
≤ 15	214 (45.9)	205 (47.7)	9 (25.0)	
16–25	161 (34.6)	152 (35.3)	9 (25.0)	
26–40	77 (16.5)	63 (14.7)	14 (38.9)	
> 40	14 (3.0)	10 (2.3)	4 (11.1)	
Trauma team activation, *n* (%)	**465**	**429**	**36**	
Yes	78 (16.8)	62 (14.5)	16 (44.4)	< 0.001
No	387 (83.2)	367 (85.5)	20 (55.6)	
Blood transfusion, *n* (%)	**466 (100)**	**430**	**36**	< 0.001
Yes	50 (10.7)	38 (8.8)	12 (33.3)	
No	416 (89.3)	392 (91.2)	24 (66.7)	
Respiratory assistance, *n* (%)	**459**	**423**	**36**	< 0.001
Yes	211 (46.0)	179 (42.3)	32 (88.9)	
No	248 (54.0)	244 (57.7)	4 (11.1)	
ICU admission, *n* (%)	**466**	**430**	**36**	< 0.001
Yes	262 (56.2)	232 (54.0)	30 (83.3)	
No	204 (43.8)	198 (46.1)	6 (16.7)	
Days in ICU (in days)				0.01
Median (IQR)	2 (0–11)	2 (0–11)	5.5 (2–12)	
Days in hospital (in days)				0.01
Median (IQR)	7 (4–19)	8 (4–20)	5.5 (2–12)	

GCS, Glasgow Coma Scale; ICU, intensive care unit; ISS, injury severity score; IQR, interquartile range

**Table 4:** Associations of patient and injury characteristics with ICU admission

Independent variable		
	ICU admission	ICU admission
	Unadjusted OR (95% CI) *P* value	Adjusted OR (95% CI) *P* value
**Age**		
Adolescent	Ref	Ref
Infant	0.30 (0.14–0.62) < 0.001	0.56 (0.12–2.46) 0.44
Preschool	0.72 (0.43–1.20) 0.21	1.75 (0.66–4.61) 0.25
School	0.90 (0.57–1.41) 0.66	1.46 (0.67–3.17) 0.33
**Mechanism of injury**		
Motor vehicle	Ref	Ref
Motorcycle	0.60 (0.25–1.39) 0.23	5.24 (1.29–21.2) 0.02
Pedal	2.41 (0.26–21.9) 0.43	15.0 (1.22–186.7) 0.03
Pedestrian	0.81 (0.47–1.42) 0.47	2.33 (0.79–6.81) 0.12
Fall > 1 m	0.46 (0.26–0.81) 0.008	2.65 (0.85–8.17) 0.09
Fall ≤ 1 m	0.31 (0.15–0.61) 0.001	5.01 (1.28–19.5) 0.02
Assault/accident	1.65 (0.51–5.35) 0.39	18.6 (3.26–106.3) < 0.001
Other	0.60 (0.14–2.46) 0.48	1.69 (0.07–37.5) 0.73
**Injury type**		
Scalp injury	Ref	
Fracture	0.30 (0.14–0.60) 0.001	0.47 (0.14–1.56) 0.21
Brain contusion	1.57 (0.86–2.86) 0.14	1.85 (0.60–5.73) 0.28
Intracranial haematoma	0.85 (0.53–1.37) 0.52	1.31 (0.60–2.87) 0.48
Other brain injuries	0.65 (0.30–1.38) 0.26	0.61 (0.14–2.53) 0.50
**Mode of arrival**		
Red Crescent ambulance Government ambulance Helicopter Private ambulance Private/police vehicle	Ref 5.08 (2.50–10.33) < 0.001 14.2 (2.73–73.79) 0.002 1.08 (0.47–2.46) 0.85 3.61 (1.26–10.33) 0.016	Ref 3.19 (1.07–9.52) 0.03 5.47 (0.44–67.4) 0.18 0.97 (0.24–3.83) 0.97 0.70 (0.12–3.93) 0.69
**ISS**	1.12 (1.09–1.15) < 0.001	1.06 (1.02–1.11) 0.003
**Trauma team activation**	2.43 (1.42–4.17) 0.001	0.69 (0.25–1.89) 0.48
**Blood transfusion**	3.06 (1.52–6.15) 0.002	1.12 (0.37–3.40) 0.83
**Respiratory assistance**	23.4 (13.8–39.8) < 0.001	25.9 (11.5–58.4) < 0.001
**Length of stay in hospital**	1.18 (1.14–1.23) < 0.001	1.14 (1.08–1.19) < 0.001
**Require operation**	2.85 (1.94–4.17) < 0.001	3.11 (1.49–6.51) 0.002

CI, confidence interval; HR, heart rate; ICU, intensive care unit; ISS, injury severity score; IQR, interquartile range; OR, odds ratio

## Discussion

This study focused on paediatric THIs using the data registry from a major trauma centre in Saudi Arabia. Over two-thirds of children included in this study were school children and adolescents, and MVC was the most common cause of injury (51.9%), with falls from ≤ 1 m more common in infants (55.8%). Both GCS and ISS results showed that severe injuries occurred in schoolchildren and adolescents, with an overall mortality rate of 7.7% and more than half of the cases admitted to the ICU. In the univariate analysis, infants were less likely to be admitted to the ICU than adolescents; however, after controlling for confounders, the ICU was associated with factors such as falls from ≤ 1 m, the ISS, length of hospital stay, respiratory assistance and the need for surgery.

In this study, most of the children who sustained head injuries were schoolchildren 6–12 years of age (37.7%), which is similar to reports from India, where head injuries are common in the same age group (43.2%) [19]. However, much of the literature has reported that head injuries are more common in children under the age of five than in other age groups [20, 21]. A study from the United Kingdom (UK) found that over 40% of traumatic injuries in infants were due to head injuries, while only 22.1% were in children aged 11–15 years [6]. According to an Australian study that included children under 16 years, more than 16.5% of head injuries occurred in infants, and over 49.0% of the total head injuries occurred in children under three years of age [22]. This was attributed to an infant’s lack of mobility control and the proportion of the head compared to body size.

The higher number of schoolchildren with THIs in our study could be explained by the fact that they are involved in more risky outdoor activities and are more independent than infants and younger children. MVCs were the most common mechanism of injury, and while infants are usually secured in a child car seat, schoolchildren may not have the same level of protection. The higher prevalence of MVCs may also be influenced by factors related to culture or the environment, such as schoolchildren spending time outside without supervision. Further investigation is needed to assess factors related to traffic safety implementation and family awareness about the importance of car restraints for children.

The GCS was used to evaluate neurological damage and the ISS for injury severity. We found that over 55.0% of the cases where GCS was applied had a minor injury, while 46.0% had minor injuries based on the ISS. These findings are similar to other reports and may be explained by the flexibility and resilience of children’s brains—especially younger children [23, 24]. We noticed an increase in injury severity as age increased in schoolchildren and adolescents, with each group accounting for 40.5% of severe injuries. This was attributed to MVCs being common among these two age groups, suggesting high-impact injuries. These findings highlight the association between age, the mechanism of injury and injury severity, and that, while younger children experience minor head injuries, the risk increases as age increases and is primarily driven by the mechanism of injury.

Mortality after a THI varies across paediatric studies [3, 25]. This is attributed to factors such as sample size, inclusion criteria, the age groups and the quality of the trauma care facility. A UK study included over 3000 THI cases and reported a mortality rate ranging from 5.8 to 7.08% over a 15-year period [6]. That study included children aged < 1 year to 15 years with moderate to severe THIs. A lower mortality rate (0.8%) was reported in an Australian study, which included over 1400 cases [26]; however, the majority of those cases were classified as mild THIs (93.0%). A Norway study that included children under 15 years reported a mortality rate of 1.8% [27]. However, that study was conducted in a trauma centre, suggesting that early intervention and the availability of specialised personnel contributed to their favourable results. The high mortality rate in our study may be attributed to the case-mix differences which consisted of a high proportion of children with severe to critical injuries (19.5%). This is further supported by the Kaplan-Meier curves, which showed higher mortality among children with lower GCS scores and those with a higher ISS. This was due to MVCs being the most common cause of injury. Additionally, two-thirds of our cases were transported via government ambulance, suggesting that these patients were likely transferred from another facility, as government ambulances primarily focus on inter-facility transfers.

The multivariate analysis revealed several significant factors associated with ICU admission, providing valuable insights into the predictors of ICU admission within the age groups. Although infants were less likely to be admitted to the ICU compared to adolescents in the univariate analysis, the association between age and ICU admission was not significant when controlling for other confounders. Regarding the mechanism of injury, motorcycle accidents were more likely to result in an ICU admission compared to MVCs. This is likely due to the lack of protective barriers and the increased risk of ejection during motorcycle crashes. Falls from ≤ 1 m were also associated with ICU admission. Interestingly, over half of the patients who suffered falls from ≤ 1 m were infants, which aligns with the literature and suggests that infants can suffer serious injuries even with a low-impact trauma cause [28].

A 6.0% increase in the odds of ICU admission was found with each unit increase in ISS. Children with severe injuries require close monitoring for neurological changes, airway and breathing support (e.g. intubation and mechanical ventilation), management for secondary complications, and post-operative care [25, 29, 30]. In our study, respiratory assistance and surgical needs were associated with ICU admission (as were longer hospital stays) highlighting that severe injuries demand advanced care and extended recovery. These findings contribute valuable data on healthcare resource utilization for paediatric trauma cases, highlighting the importance of ICU capacity, surgical intervention, and respiratory support, which are essential for managing the high-acuity needs of this patient population.

There are some limitations to this study. As the data was collected retrospectively, we could not control for all possible confounders, which could have affected our results and conclusion. In addition, it was difficult to establish a precise temporal relationship between variables. Furthermore, the reliance on previously recorded data introduced the possibility of incomplete or inconsistent data, which may have affected the accuracy of our findings. This study was based on a single centre in Saudi Arabia; therefore, the findings may not represent all paediatric populations. Information regarding the use of protective measures such as seat belts in MVCs and helmets in motorcycle and pedal crashes was not available in the STAR database, which could be crucial for understanding their impact on injury severity and outcomes. There was a discrepancy in the documentation of trauma team activation for cases of severe head trauma (GCS 3–8). Although King Saud Medical City adheres to internationally validated policies regarding Trauma team activation, Table 3 illustrates that only 78 of the 147 severe head trauma cases were documented to have trauma team activation. The discrepancy is due to an error or limitation in the data collectors’ documentation in the STAR database. Further, we did not include other variables such as pupillary reaction, time from injury to ED arrival etc. in this analysis as this data was not available. We also did not consider injury severity markers, that might be available at a later stage based on imaging such as Rotterdam CT-scan score, because the data is intended to inform patterns of injuries and trajectories of patient groups at the point of admission to the ED. The sample size was moderate, which affected the generalisability of the study and reduced the statistical power, especially after categorising paediatric ages into four groups. However, despite this, the sample size was sufficient to identify significant associations between key variables such as injury severity, mortality and ICU admission. We also chose not to perform a logistic regression model for mortality due to the small number of mortality cases (*n* = 36) and concerns over model overfitting. Data on long-term outcomes were not available, which is particularly important given that children are known to be at risk for long-term disabilities following a THI. However, despite these limitations, this data provides a valuable benchmark for trauma systems across Saudi Arabia and similar regions, helping inform standards for performance and outcomes in paediatric trauma care.

## Conclusions

The majority of children who suffered from THIs were schoolchildren and adolescents. Injury cause and type varied by age group. Falls were common in infants, while MVCs were more pronounced in older children. Injury severity was associated with mortality and ICU admission, suggesting its strength in predicting outcomes following a paediatric THI.

Future efforts should focus on enhancing prevention strategies tailored to age-specific risks. Further research is needed to explore the long-term outcomes of paediatric THIs across different age groups and assess the effectiveness of targeted interventions to reduce injury severity. Multi-centre studies with larger sample sizes could provide more generalisable data and help validate our findings.

## Data Availability

The datasets used during the current study are available from the corresponding author at reasonable request and with permission from the IRB, KSMC, Riyadh, Saudi Arabia.

## Abbreviations

aOR: Adjusted odds ratio
BP: Blood pressure
CI: Confidence interval
ED: Emergency department
GCS: Glasgow Coma Scale
HR: Heart rate
ISS: Injury severity score
ICU: Intensive care unit
IQR: Interquartile range
KSMC: King Saud Medical City
MVC: Motor vehicle crash
OR: Odds ratio
RR: Respiratory rate
RTA: Road traffic accident
STAR: Saudi TraumA Registry
THI: Traumatic head injury
UK: United Kingdom
